# Supplementary material for: Approximate distance correlation for selecting highly interrelated genes across datasets
Source: PLoS Comput Biol. 2021 Nov 9;17(11):e1009548. doi: 10.1371/journal.pcbi.1009548 (PMC8604336; doi:10.1371/journal.pcbi.1009548)
Supplement: S1 Supplementary Materials — Further detailed descriptions of the principle of ADC, the computational complexity of ADC, and more information about the methods and process of data analysis for single-cell RNA-seq data. Fig A. Simulation experiments on DC combined with the BH method in terms of Power and FDR (target is 20%). We generated each pair of variables with 3000 and 10,000 dimensions, respectively. Every non-zero entry of the variables was sampled from a beta(2,4) distribution. (a) Each pair of vectors are dense and k dimensions are shared with a linear transform. (b) Each pair of vectors are sparse with 90% zero entries and k dimensions are shared with a linear transform. (c) Each pair of vectors are dense and k dimensions are shared with a log transform. (d) Each pair of vectors are sparse with 90% zero entries and k dimensions are shared with a log transform. Fig B. Performance of ADC on simulated datasets. (a and b) Running time and peak menmory cost of ADC with two datasets with 10 thounsand cells and different number of genes. GB indicates the GigaByte. (c) We applied ADC to data1 and data2 generated by splatter (Fig 3A) under each k from 20 to 40. For each k, we selected top 100 interrelated genes and calculated the number of overlapped top genes for each pair of k. The result is showed in the boxplot. Fig C. Functional enrichments of selected genes between five pairs of cancers. (a) BLCA and LUSC, (b) GBMLGG and LGG, (c) KIRC and KIRP, (d) STAD and STES, and (e) COAD and COADRED. Fig D. PCA plots of hematopoietic stem cells CMP, GMP and MEP. (a) The cells are colored by the cell types annotated by the combination of molecular surface markers. (b) The cells are colored by the cell types annotated by an unsupervised clustering method Leiden. Fig E. Numbers of selected genes across five technologies for the data with different number highly variable genes. (a) top 2000 genes, (b) top 3000 genes, (c) top 4000 genes, and (d) top 5000 genes. Unsupervised hierarchical clustering analysi [file pcbi.1009548.s001.pdf]

Supplemental Materials for "Approximate distance correlation for selecting highly interrelated genes across datasets"

Qunlun Shen and Shihua Zhang

Abstract

This text gives detailed descriptions of the principle of ADC, the computational complexity of ADC, and more information about the methods and process of data analysis for single cell RNA-seq. Figures supplemented to the main text and tables served as brief descriptions to datasets are also included.

1. Supplemental Methods

1.1. Distance covariance and distance correlation

Distance covariance is a method to measure the distance between the product of marginal characteristic functions of two random vectors $X \in R^m$, $Y \in R^n$, and their joint characteristic function [1]. It is defined as:

$$\begin{aligned}\mathcal{V}^2(X,Y) &= \|\phi_{X,Y}(t,s) - \phi_X(t)\phi_Y(s)\|_w^2 \\ &= \int_{\mathbb{R}^{m+n}} |\phi_{X,Y}(t,s) - \phi_X(t)\phi_Y(s)|^2 w(t,s)dtds,\end{aligned} \tag{1}$$

where

$$w(t,s) = \left(c_m c_n |t|_n^{1+n} |s|_m^{1+m}\right)^{-1}, \quad c_d = \frac{\pi^{\frac{1+d}{2}}}{\Gamma\left(\frac{1+d}{2}\right)}, \tag{2}$$

$\Gamma(\cdot)$ is the gamma function and the weight function $w(t,s)$ ensures distance covariance less than infinity, ϕ_X, ϕ_Y and $\phi_{X,Y}$ denote the characteristic functions of X, Y and (X,Y) . This definition is similar to that of the classical covariance and has a significant property that the X and Y are independent if and only if $\mathcal{V}^2(X,Y) = 0$.

As the standard definition of correlation coefficient, the distance correlation (DC) is defined as:

$$\mathcal{R}^2(X,Y) = \frac{\mathcal{V}^2(X,Y)}{\sqrt{\mathcal{V}^2(X,X)\mathcal{V}^2(Y,Y)}}. \tag{3}$$

However, we don't know the exact distribution of X and Y, we just have some observations of them, so we will use sample estimation to approximate DC. Let ϕ_X^k, ϕ_Y^k and $\phi_{X,Y}^k$ denote the empirical characteristic functions of X, Y and (X,Y). The sample distance covariance for random vectors X, Y is defined as:

$$\mathcal{V}_k^2(X,Y) = \left\|\phi_{X,Y}^k(t,s) - \phi_X^k(t)\phi_Y^k(s)\right\|_w^2, \tag{4}$$

where

$$\left\|\phi_{x,y}^k(t,s) - \phi_x^k(t)\phi_y^k(s)\right\|_w^2 = \int_{\mathbb{R}^{m+n}} \left|\phi_{x,y}^k(t,s) - \phi_x^k(t)\phi_Y^k(s)\right|^2 w(t,s)dtds, \tag{5}$$

and the weight function $w(t,s)$ is the same as Eq. (2).

Given k random samples of observations $(X^i, Y^i) \in R^{m+n}$, $i = \{1, \ldots, k\}$, let's define

$$\begin{aligned} A_{i,j} &= \left|X^i - X^j\right| - \frac{1}{k}\sum_{v=1}^{k}\left|X^v - X^j\right| - \frac{1}{k}\sum_{u=1}^{k}\left|X^i - X^u\right| + \frac{1}{k^2}\sum_{v,u=1}^{k}\left|X^v - X^u\right|, \\ B_{i,j} &= \left|Y^i - Y^j\right| - \frac{1}{k}\sum_{v=1}^{k}\left|Y^v - Y^j\right| - \frac{1}{k}\sum_{u=1}^{k}\left|Y^i - Y^u\right| + \frac{1}{k^2}\sum_{v,u=1}^{k}\left|Y^v - Y^u\right|, \end{aligned} \tag{6}$$

$i, j = 1, \ldots, n$, and $|\cdot|$ denotes the Euclidean norm. Let

$$\begin{aligned} &a_{ij} = \left|X^i - X^j\right|, \quad i, j = 1, \ldots, k, \\ &a_{i\cdot} = \sum_{j=1}^{k} a_{ij}, \quad a_{\cdot j} = \sum_{i=1}^{k} a_{ij}, \quad \bar{a}_i = \bar{a}_{i\cdot} = \frac{1}{k} a_{i\cdot}, \\ &a_{\cdot\cdot} = \sum_{i,j=1}^{k} a_{ij}, \quad \bar{a} = \frac{1}{k^2}\sum_{i,j=1}^{k} a_{ij}, \end{aligned} \tag{7}$$

and similarly b_{ij}, $b_{i\cdot}$, $b_{\cdot j}$, $\bar{b}_i$, $b_{\cdot\cdot}$, $\bar{b}$ for Y.

We can calculate the distance covariance $\mathcal{V}_k^2(X, Y)$ as follows

$$\mathcal{V}_k^2(X, Y) = \frac{1}{k(k-3)}\left\{\sum_{i,j=1}^{k} A^*_{i,j} B^*_{i,j} - \frac{k}{k-2}\sum_{i=1}^{k} A^*_{i,i} B^*_{i,i}\right\}, \tag{8}$$

where

$$A^*_{i,j} = \begin{cases} \frac{k}{k-1}\left(A_{i,j} - \frac{a_{ij}}{k}\right), & i \neq j, \\ \frac{k}{k-1}\left(\bar{a}_i - \bar{a}\right), & i = j, \end{cases} \quad B^*_{i,j} = \begin{cases} \frac{k}{k-1}\left(B_{i,j} - \frac{b_{ij}}{k}\right), & i \neq j, \\ \frac{k}{k-1}\left(\bar{b}_i - \bar{b}\right), & i = j, \end{cases} \tag{9}$$

and the distance correlation is

$$DC(X, Y) = \mathcal{R}_k^2(X, Y) = \frac{\mathcal{V}_k^2(X, Y)}{\sqrt{\mathcal{V}_k^2(X, X)\mathcal{V}_k^2(Y, Y)}}. \tag{10}$$

It can be proved that under the independence hypothesis, as p, q tend to infinity

$$\mathcal{T}_k = \sqrt{v-1} \cdot \frac{\mathcal{R}_k^2}{\sqrt{1 - \left(\mathcal{R}_k^*\right)^2}} \tag{11}$$

converges to t-distribution with $\frac{k(k-3)-2}{2}$ degrees of freedom, so we can calculate the p-value easily for each hypothesis testing.

1.2. The ADC Algorithm

Given two gene expression datasets $\mathbf{X}$ and $\mathbf{Y}$ with matched genes (if genes are not matched, we use their common genes as input), we use MG (matched genes) to denote the genes of these datasets and use HIG (highly interrelated genes) to denote the genes selected by ADC. We calculate the p-value of the distance correlation for each gene between the two datasets. However, for each pair of genes, we only have one observation, this is just like we have only one observation in linear regression, which not only leads to be incalculable but also lose statistical power. In order to overcome this issue, for each gene in a single dataset, we select the most relevant k genes to it (measured by Pearson correlation) as its k approximate observations, the matrix constructed by the expression of the above k genes is called the approximate observations for this gene. Since many genes show similar expression in a single experiment, this approximation is reasonable from the biological view. The value of k is set to 30 by default.

Algorithm 1 Approximate observation searching (AOS)

Input:

Two gene expression datasets $\mathbf{X}$ and $\mathbf{Y}$ with matched genes.

Output:

Genes with their approximate observations in each dataset.

```
  X_cor ← Pearson correlation matrix for genes in X.
  Y_cor ← Pearson correlation matrix for genes in Y.
for all gene ∈ MG do
  AOS_X(gene) ← k most correlated genes with gene in dataset X
  AOS_Y(gene) ← k most correlated genes with gene in dataset Y
end for
```

Algorithm 2 ADC

Input:

Two gene expression datasets $\mathbf{X}$ and $\mathbf{Y}$ with matched genes, the FDR level.

Output:

Genes with similar expression between two datasets.

```
for all gene ∈ MG do
  X_gene ← AOS_X(gene).
  Y_gene ← AOS_Y(gene).
end for
for all gene ∈ MG do
  Calculate the p-value of distance correlation of X_gene and Y_gene.
end for
  Use the BH method to achieve the adjusted p-value.
  HIG ← genes with the adjusted p-value less than the FDR level.
return HIG
```

1.3. The computational complexity of distance correlation

Given two random vectors X and Y of m and n dimensions respectively, we use k to denote the observations of these two vectors. The complexity of calculating all the A_{ij} and B_{ij} in Eq. (6) is $O(k^2) + O(k\max\{m,n\})$. We can see that the complexity of calculating A^*_{ij} is just the same as A_{ij} in Eq. (9). After that, we need to calculate $\mathcal{V}^*_k(X,Y), \mathcal{V}^2_k(X,X)$ and $\mathcal{V}^2_k(Y,Y)$, the complexity of each is the same. We can deduce from Eqs. (8) and (9) that the complexity is $O(k^2(k^2 + k\max\{m,n\})) = O(k^3(k + \max\{m,n\}))$. The complexity of calculating distance correlation coefficient and p-value is also $O(k^3(k + \max\{m,n\}))$.

1.4. The scRNA-seq simulation data generated by splatter

We used the splatSimulate function in the R package splatter [2] to simulate scRNA-seq datasets with different cell types. Each dataset was generated with 1000 cells and 5000 genes. For data1, data2 and data3, the grop.prob was set as c(0.80,0.20,0,0), c(0, 0.40, 0.60, 0), and c(0.60, 0.40, 0, 0), respectively. Method was set as "groups", and other parameters was set as the default.

1.5. Running time and peak memory profiling

Simulated datasets were generated for this testing. Specifically, each entry of the data matrix was sampled from a standard uniform distribution. We used the time module and mprof module of python to obtain the running time and peak memory cost, respectively. The memory cost of the raw data measures that of generating the data by the numpy.random.random() function in python. All the methods were run on a 2.30 GHz Intel Xeon E5-26680 v2 central processing unit with 368 GB of RAM.

1.6. Enrichment analysis of the selected genes

Enrichment analysis was done by enrichGO function from R package clusterProfiler [3]. We detected enriched go terms based on the hypergeometric test, and the p-values were adjusted by FDR (FDR$<$0.05) to prevent high false discovery rate. The top ten enriched functional terms were shown.

1.7. Functional network analysis of the selected genes

We analyzed the functional network of the top selected genes by GeneMANIA [4]. The input genes were showed in red colour and top 10 related genes were added into the network which were showed in white colour. Physical interactions and genetic interactions were showed with different coloured edges. The size of the node is proportional to its degree, and the network was plotted by Cytoscape [5]. Unless carefully specified, we use top 100 highly interrelated genes selected by ADC as the input, the subnetwork which only contains less than 3 genes were removed in the output of GeneMANIA.

1.8. Heatmap and hierarchical clustering

We used heatmap to show the number of the selected genes between different datasets, and further used the reciprocal value of that number to construct the distance matrix as the input for unsupervised hierarchical clustering analysis. The clustering was done by scipy.cluster.hierarchy.linkage in a python package scipy [6].

1.9. Highly variable genes selection

We selected highly variable genes [7] using the scanpy.pp.highly_variable_genes in scanpy with default parameters in pancreatic islet cells and hematopoietic progenitors.

1.10. Cross-species analysis

We applied ADC to the hematopoietic stem cells (HSCs) data between human [8] and mouse [9] to find tissue-specific conserved genes. The annotation information of homologous genes was obtained by R package biomaRt]10]. Cells with less than 200 expressed genes and genes expressed in less than 3 cells were filtered. Also, cells with more than 25% mitochondrial genes present were filtered. After that, we normalized the gene counts per cell with the scanpy package and log-transformed the expression with a pseudo-count 1 and the z-score normalization was also performed for each gene. ADC returned 232 highly interrelated genes where FDR was set as 0.01. The enrichment analysis was done by Metascape [11], which shows that these genes capture conserved functions between human and mouse, and these functions also exist in HSCs (Fig F in S1 Supplementary Materials). Specifically, signaling by GPCR (G protein-coupled receptor) is the top one enriched function, where GPCRs are widely expressed in human and mouse [12]. Also, GPCRs are wildly expressed in HSCs [13].‘

References

[1] GáBor J SzéKely and Maria L Rizzo. The distance correlation t-test of independence in high dimension. *Journal of Multivariate Analysis*, 117:193–213, 2013.

[2] Luke Zappia, Belinda Phipson, and Alicia Oshlack. Splatter: simulation of single-cell rna sequencing data. *Genome biology*, 18(1):174, 2017.

[3] Guangchuang Yu, Li-Gen Wang, Yanyan Han, and Qing-Yu He. clusterprofiler: an r package for comparing biological themes among gene clusters. *Omics: a journal of integrative biology*, 16(5):284–287, 2012.

[4] Sara Mostafavi, Debajyoti Ray, David Warde-Farley, Chris Grouios, and Quaid Morris. Genemania: a real-time multiple association network integration algorithm for predicting gene function. *Genome biology*, 9(S1):S4, 2008.

[5] Paul Shannon, Andrew Markiel, Owen Ozier, Nitin S Baliga, Jonathan T Wang, Daniel Ramage, Nada Amin, Benno Schwikowski, and Trey Ideker. Cytoscape: a software environment for integrated models of biomolecular interaction networks. *Genome research*, 13(11):2498–2504, 2003.

[6] Pauli Virtanen, Ralf Gommers, Travis E Oliphant, Matt Haberland, Tyler Reddy, David Cournapeau, Evgeni Burovski, Pearu Peterson, Warren Weckesser, Jonathan Bright, et al. Scipy 1.0: fundamental algorithms for scientific computing in python. *Nature methods*, 17(3):261–272, 2020.

[7] F Alexander Wolf, Philipp Angerer, and Fabian J Theis. Scanpy: large-scale single-cell gene expression data analysis. *Genome biology*, 19(1):15, 2018.

[8] Danilo Pellin, Mariana Loperfido, Cristina Baricordi, Samuel L Wolock, Annita Montepeloso, Olga K Weinberg, Alessandra Biffi, Allon M Klein, and Luca Biasco. A comprehensive single cell transcriptional landscape of human hematopoietic progenitors. *Nature communications*, 10(1):1–15, 2019.

[9] Sonia Nestorowa, Fiona K Hamey, Blanca Pijuan Sala, Evangelia Diamanti, Mairi Shepherd, Elisa Laurenti, Nicola K Wilson, David G Kent, and Berthold Göttgens. A single-cell resolution map of mouse hematopoietic stem and progenitor cell differentiation. *Blood, The Journal of the American Society of Hematology*, 128(8):e20–e31, 2016.

[10] Damian Smedley, Syed Haider, Benoit Ballester, Richard Holland, Darin London, Gudmundur Thorisson, and Arek Kasprzyk. Biomart–biological queries made easy. *BMC genomics*, 10(1):1–12, 2009.

[11] Yingyao Zhou, Bin Zhou, Lars Pache, Max Chang, Alireza Hadj Khodabakhshi, Olga Tanaseichuk, Christopher Benner, and Sumit K Chanda. Metascape provides a biologist-oriented resource for the analysis of systems-level datasets. *Nature communications*, 10(1):1–10, 2019.

[12] Demetrios K Vassilatis, John G Hohmann, Hongkui Zeng, Fusheng Li, Jane E Ranchalis, Marty T Mortrud, Analisa Brown, Stephanie S Rodriguez, John R Weller, Abbie C Wright, et al. The g protein-coupled receptor repertoires of human and mouse. *Proceedings of the National Academy of Sciences*, 100(8):4903–4908, 2003.

[13] Robert Möhle and Adriana C Drost. G protein–coupled receptor crosstalk and signaling in hematopoietic stem and progenitor cells. *Annals of the New York Academy of Sciences*, 1266(1):63–67, 2012.

2. Supplemental Figures

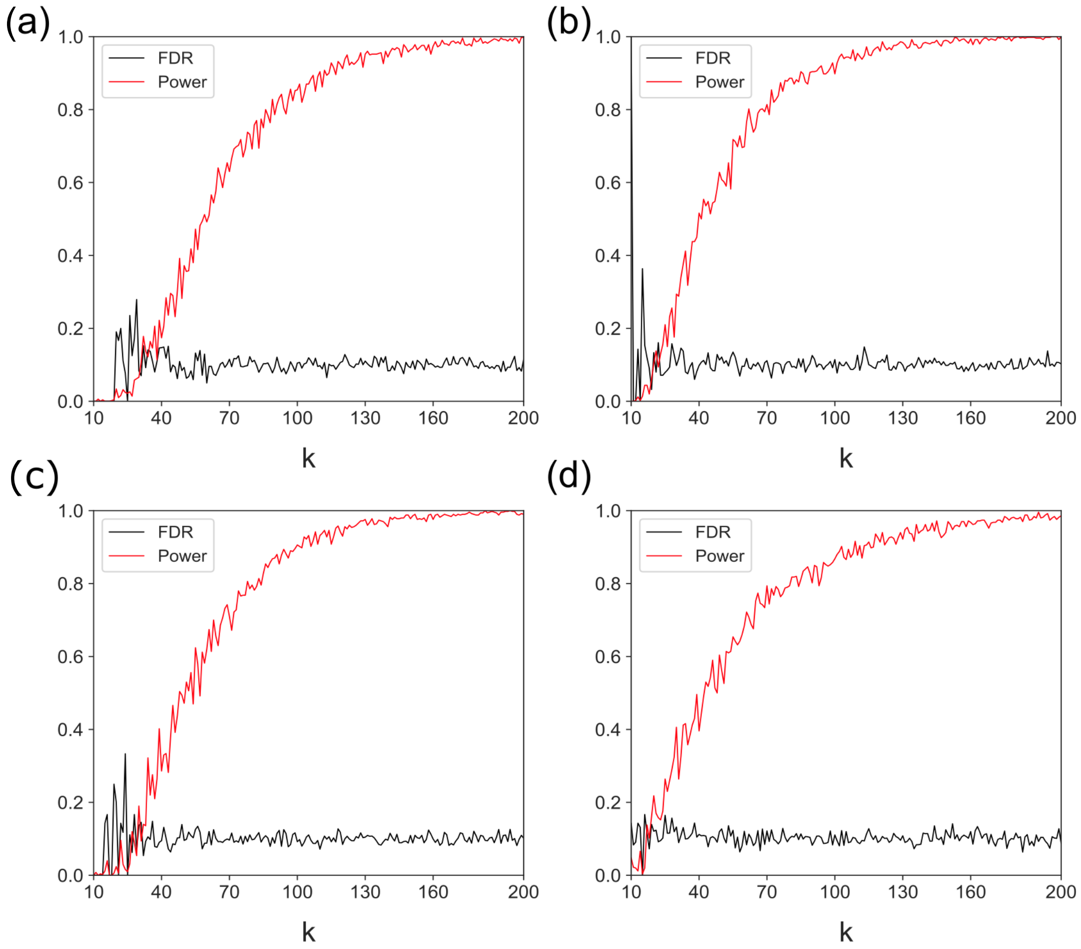

Fig A. Simulation experiments on DC combined with the BH method in terms of Power and FDR (target is 20%). We generated each pair of variables with 3000 and 10,000 dimensions, respectively. Every non-zero entry of the variables was sampled from a beta(2,4) distribution. (a) Each pair of vectors are dense and k dimensions are shared with a linear transform. (b) Each pair of vectors are sparse with 90% zero entries and k dimensions are shared with a linear transform. (c) Each pair of vectors are dense and k dimensions are shared with a log transform. (d) Each pair of vectors are sparse with 90% zero entries and k dimensions are shared with a log transform.

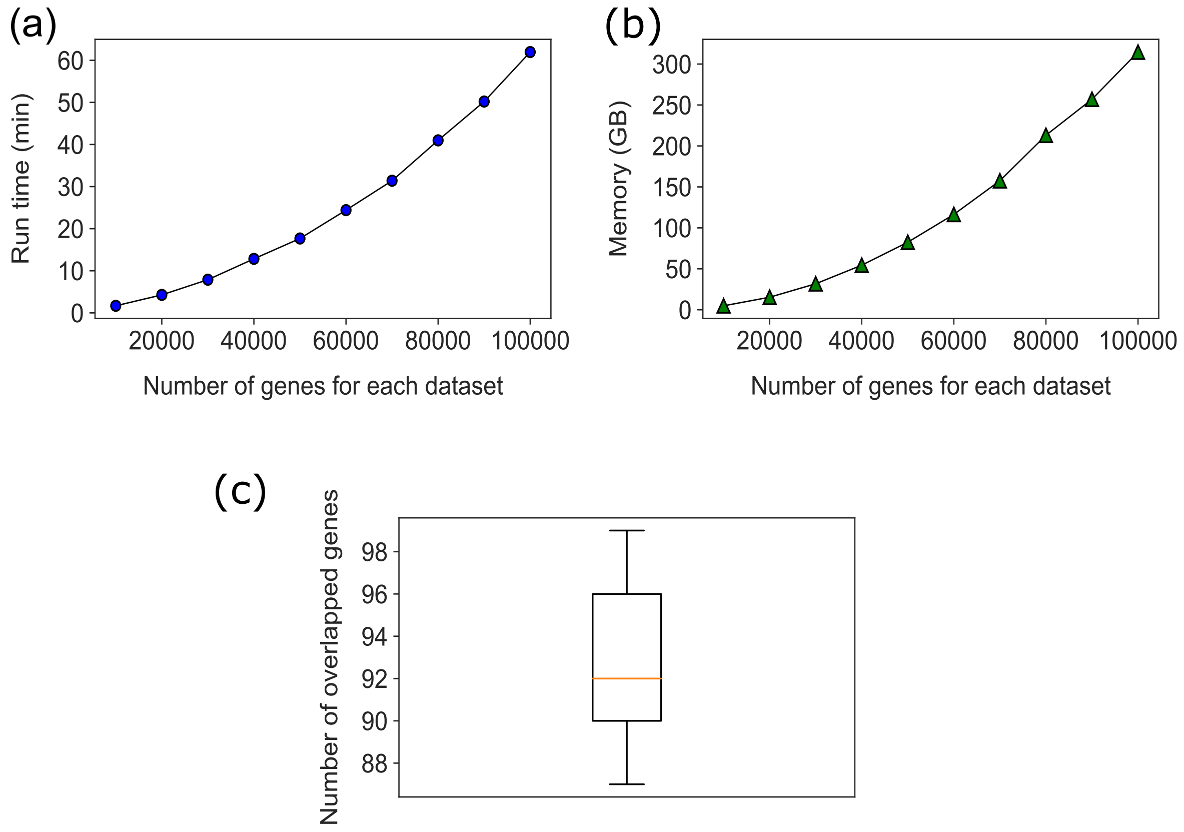

Fig B. Performance of ADC on simulated datasets. (a and b) Running time and peak memory cost of ADC on two datasets with 10 thousand cells and different number of genes. GB indicates the GigaByte. (c) Boxplot of the numbers of the overlapped genes between the top 100 highly interrelated genes by applying ADC to data1 and data2 (generated by splatter in Fig. 3A) for each pair of ks ranging from 20 to 40.

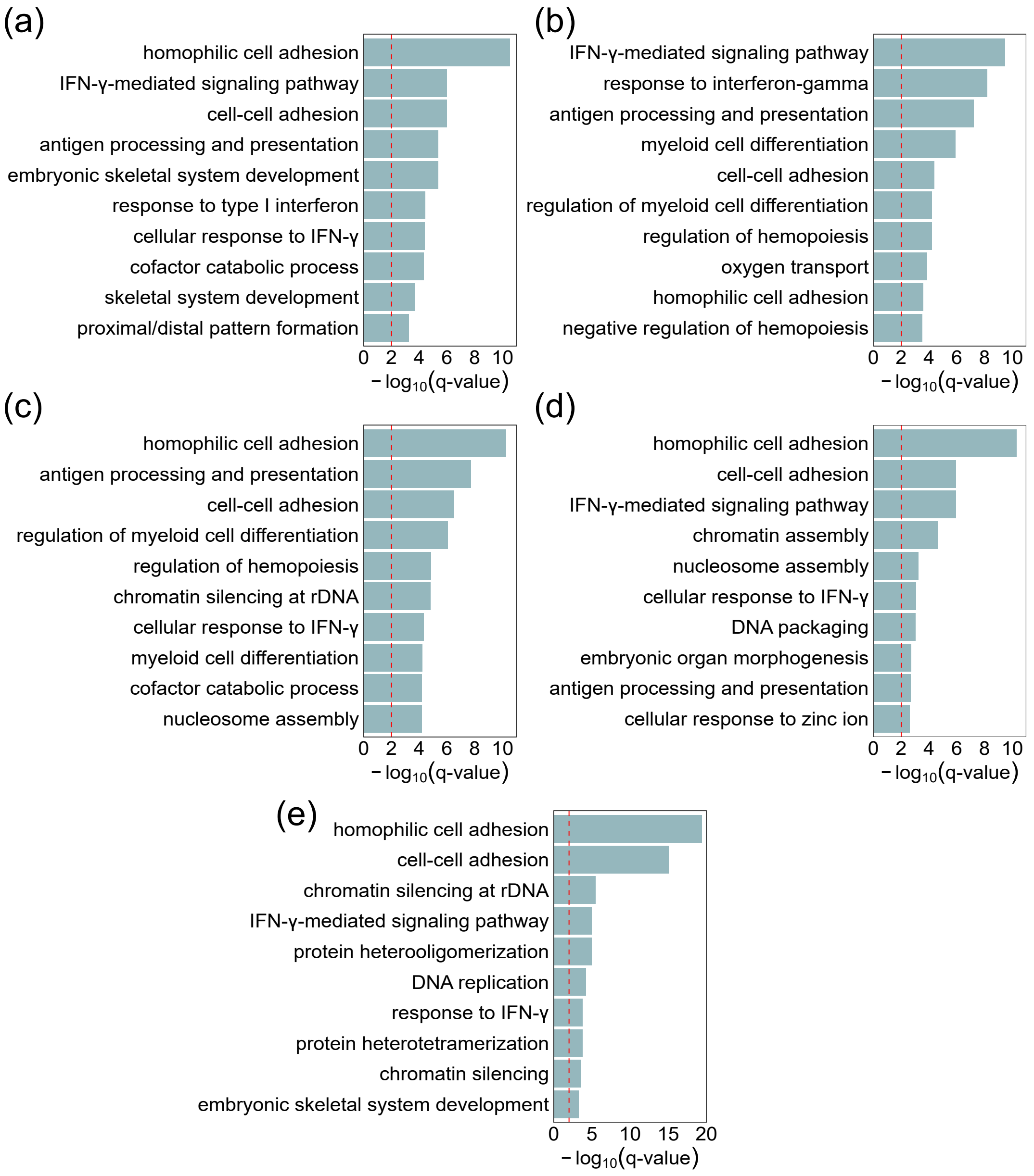

Fig C. Functional enrichments of selected genes between five pairs of cancers including (a) BLCA and LUSC, (b) GBMLGG and LGG, (c) KIRC and KIRP, (d) STAD and STES, and (e) COAD and COADRED.

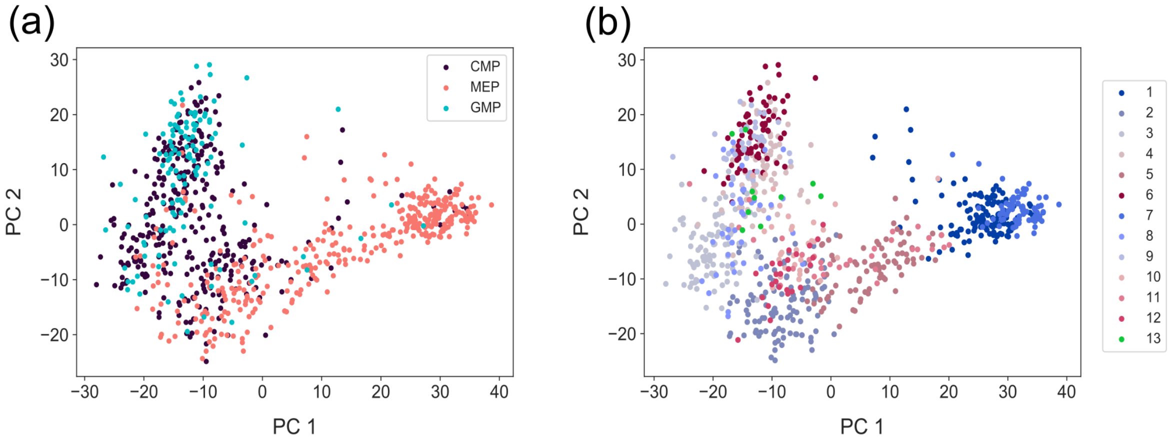

Fig D. PCA plots of hematopoietic stem cells CMP, GMP and MEP. (a) The cells are colored by the cell types annotated by the combination of molecular surface markers. (b) The cells are colored by the cell types annotated by an unsupervised clustering method Leiden.

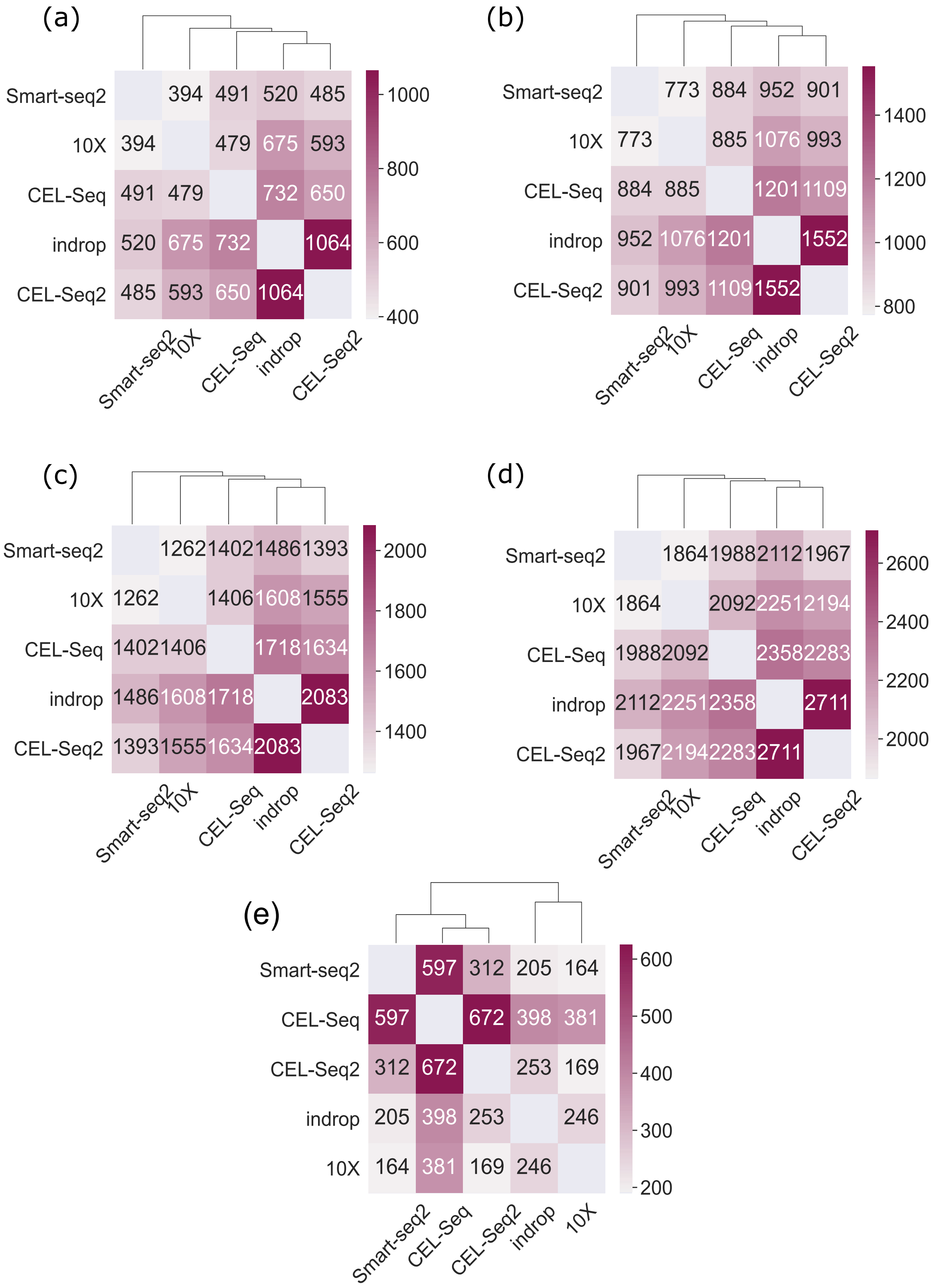

Fig E. Numbers of selected genes across five technologies for the data with different number highly variable genes: (a) top 2000 genes, (b) top 3000 genes, (c) top 4000 genes, and (d) top 5000 genes. (e) Highly interrelated genes selected by the ensemble version of ADC. Unsupervised hierarchical clustering analysis is performed with the reciprocal value of the numbers.

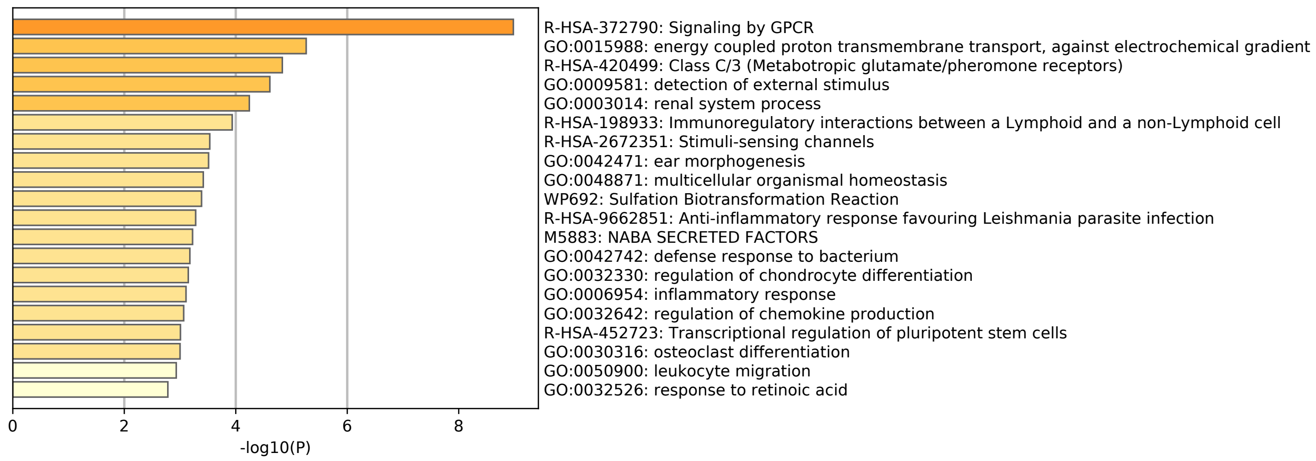

Fig F. Enriched GO terms of highly interrelated genes between human and mouse done by Metascape.

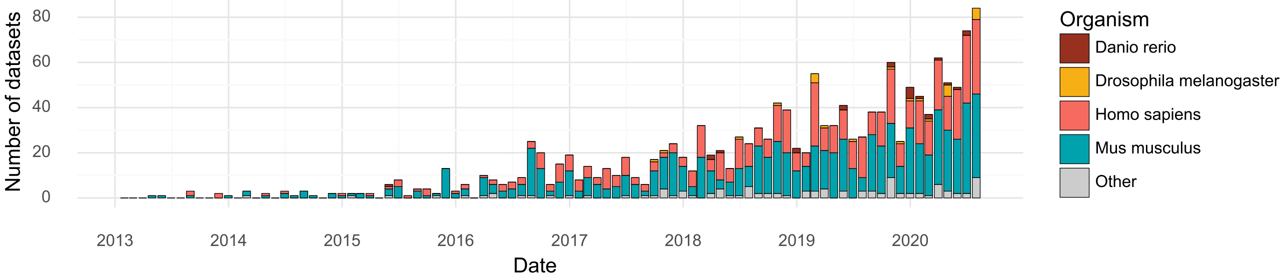

Fig G. Number of scRNA-seq datasets per month from January 2013 to July 2020. The statistics was obtained from NCBI Gene Expression Omnibus with key words: "single cell RNA seq" or "single cell transcriptome" or "single cell gene expression".

3. Supplemental Tables

Table A. Number of samples of 21 types of cancer. 17 out of 38 types of cancer in TCGA were excluded in our study due to limited numbers of samples.

Cancer Type		Tumor	Normal	Total
**BLCA**	Bladder Urothelial Carcinoma	408	19	427
**BRCA**	Breast Invasive Carcinoma	1100	112	1212
**CESC**	Cervical Squamous Cell carcinoma and endocervical adenocarcinoma	306	3	309
**COAD**	Colon Adenocarcinoma	459	41	500
**COADREAD**	Colon Adenocarcinoma Rectum Adenocarcinoma Esophageal Carcinoma	626	51	677
**GBMLGG**	Glioma	696	5	701
**HNSC**	Head And Neck Squamous Cell Carcinoma	522	44	566
**KIRC**	Kidney Renal Clear Cell Carcinoma	534	72	606
**KIRP**	Kidney Renal Papillary Cell Carcinoma	291	32	323
**LGG**	Brain Lower Grade Glioma	530	0	530
**LIHC**	Liver Hepatocellular Carcinoma	373	50	423
**LUAD**	Lung Adenocarcinoma	517	59	576
**LUSC**	Lung Squamous Cell Carcinoma	501	51	552
**OV**	Ovarian Serous Cystadeno Carcinoma	307	0	307
**PRAD**	Prostate Adenocarcinoma	498	52	550
**SARC**	Sarcoma	263	2	265
**SKCM**	Skin Cutaneous Melanoma	472	1	473
**STAD**	Stomach Adenocarcinoma	415	35	450
**STES**	Stomach And Esophageal Carcinoma	600	46	646
**THCA**	Thyroid Carcinoma	509	59	568
**UCEC**	Uterine Corpus Endometrioid Carcinoma	546	34	580

Table B. Number of cells of six hematopoietic cell types.

Cell type		Cell number
**HSC**	hematopoietic stem cells	408
**CMP**	common myeloid progenitors	1100
**GMP**	granulocyte macrophage progenitors	306
**MEP**	megakaryocyte-erythroid progenitors	459
**MPP**	multipotent progenitors	626
**LMPP**	lymphoid-primed multipotent progenitor	696

Table C. Number of pancreatic islet cells from five different technologies.

Technology	Cell number
**indrop**	8569
**CEL-seq2**	4776
**10X**	2477
**CEL-seq**	1538
**Samrt-seq2**	3354

Table D. Numbers of PBMC cells from two different sequencing methods.

Sequencing method	Cell number
**scRNA-seq**	8287
**scATAC-seq**	2638
