# Supplementary material for: Approximate distance correlation for selecting highly interrelated genes across datasets
Source: PLoS Comput Biol. 2021 Nov 9;17(11):e1009548. doi: 10.1371/journal.pcbi.1009548 (PMC8604336; doi:10.1371/journal.pcbi.1009548)
Supplement: Attachment — Submitted filename: ReviewReport_ADC_v5.pdf [file pcbi.1009548.s002.pdf]

Response to Reviewer #1

In this study, the authors developed a method to study the gene expression conservation of each gene between different datasets based on distance correlation using co-expressed genes. They showed that their method can identify conserved genes from bulk RNA-seq, scRNA-seq, and scATAC-seq datasets. Although the results are compelling, the paper can be further improved. See detailed comments below.

Response: We appreciate very much for your kind recognition and suggestions. In light of your comments, we have carefully revised our manuscript.

1. The authors select k=30 highly correlated genes in each dataset to calculate the distance correlation. How the selection of k affects the result is not clear. Why selecting k=30 as the default value has to be justified.

Response: We have performed a simulation study to justify the selection of k (page 8, line 218-222): "We also test the influence of hyper-parameter k by applying ADC to Data 1 and Data 2. Here, we selected the top 100 similarly expressed gene under each k varying from 20 to 40. For each pair of k, the number of overlapped genes is calculated. In average, we got almost 93 genes, that suggests ADC is a robust algorithm considering the selection of k (Supplementary Fig. S2C)."

In light of your comments, we also proposed a more robust process to select the similarly expressed genes. Specifically, we detected the similarly expressed gene under each k varying from 20 to 40, and we kept genes which were considered as similarly expressed ones under 21 different ks. We got very consistent result on real biological applications as the original process (e.g., for the "Similarly correlated genes across modalities reveal biological relevant genes" applications in Fig. 6B and Supplementary Fig. S5E).

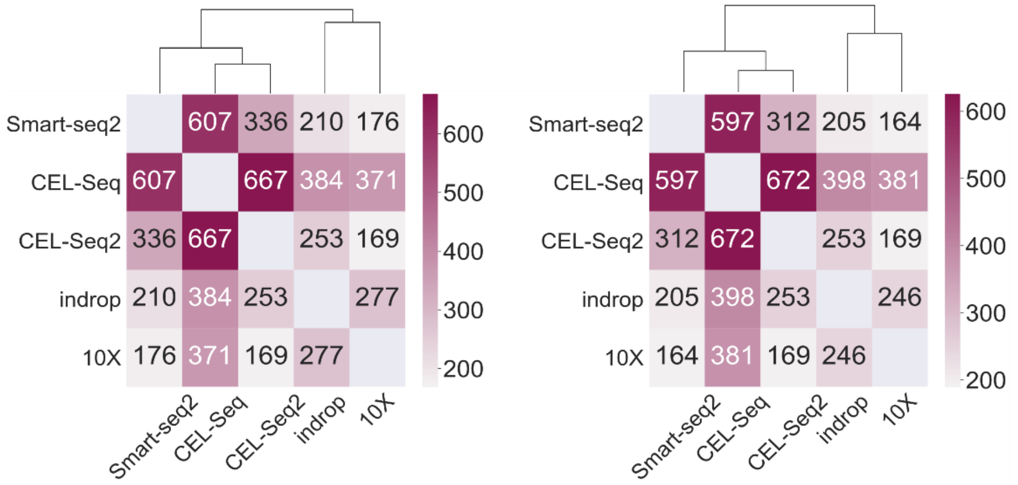

Fig. 6B and Supplementary Fig. S5E. Similarly expressed gene selected by ADC with k=30 (left) and an ensemble version (right) between each pair of these datasets (FDR=0.01). Unsupervised hierarchical clustering was performed with the reciprocal value of the number in both situations.

To clarify this, we added some sentences to the conclusion (Page 14, line 332-339).

"The number k of selected highly correlated genes in each dataset is an important parameter which could affect the gene sets for DC calculation. We have empirically demonstrated the default one work well in the simulation studies and biological applications. Alternatively, we could provide a more robust version by leveraging the similarly expressed genes selected by ADC with different ks. Specifically, we detected the similarly expressed gene under each k varying from 20 to 40, and we kept genes which were considered as similarly expressed ones under 21 different ks. The results are very consistent with the original ones (Fig. 6B and Supplementary Fig. S5E)."

2. How batch effects affect the result is not examined. The authors showed in Figure 6 that their method can capture more similar genes between sequencing platforms that are similar. It shows batch effects (here platform effects) are clearly affecting the results.

Response: This is a good point. Different platforms should have platform effects. Our purpose is just to adopt this application to demonstrate that ADC can capture more similar patterns among the data of the related platforms. This indeed verify ADC is effective. In reality, one can adopt some batch correction methods such as MNN (Haghverdi, 2018) to remove platform effects and then apply ADC further.

[1] Haghverdi L, Lun A T L, Morgan M D, et al. Batch effects in single-cell RNA-sequencing data are corrected by matching mutual nearest neighbors[J]. Nature biotechnology, 2018, 36(5): 421-427.

3. The proposed method aims to reveal genes that have similar expression patterns between two datasets. Can it be generalized to more than two datasets? For example, can it be applied to reveal common genes across all cancer samples showed in Figure 4?

Response: ADC was designed based on the distance correlation. Its principle is that two random variables X and Y are independent if and only if $\phi_X(t)\phi_Y(s) = \phi_{X,Y}(t,s)$. Then we need to estimate a joint distribution of two high dimension variables. The distance correlation is calculated based on distance covariance formulated as follows:

$$\left|\left|\phi_{X,Y}(t,s) - \phi_X(t)\phi_Y(s)\right|\right|_w = \int \left|\phi_{X,Y}(t,s) - \phi_X(t)\phi_Y(s)\right|^2 w(t,s)dtds.$$

Obviously, we need to find a suitable $w(t,s)$ to make $\left|\left|\phi_{X,Y}(t,s) - \phi_X(t)\phi_Y(s)\right|\right|_w$ calculable, i.e., less than infinity. For more than two datasets, we not only need to estimate a joint distribution of multiple high dimensional variables and but also need to find a new w function to make the distance covariance calculable. So, it is not that direct to make ADC be generalized to more than two datasets. This is reasonable that for correlation metrics like PCC are usually for pairwise random variables not for multiple ones directly.

4. Although the method is designed to reveal genes that are similar between datasets, can it reveal genes that are most diverse (not similar) between datasets?

Response: Yes, you are right. ADC (and DC) is a kind of correlation metric. It reflects the association strength of two genes here. The most significant ones capture the most similar biological signals. By contrast, the weakest ones reveal the most diverse ones.

5. For the hematopoietic single-cell RNA-seq analysis in Figure 5B, the author only showed results for CMP, GMP, and MEP for human bone marrow. How about the results from other cell types? Is it also consistent with the mouse data?

Response: In light of your suggestion, we also showed the results from other cell types including HSC, CMP, GMP, MEP, LMPP for human bone marrow dataset. They are also consistent with those of the mouse data. We added this results into Fig 5B.

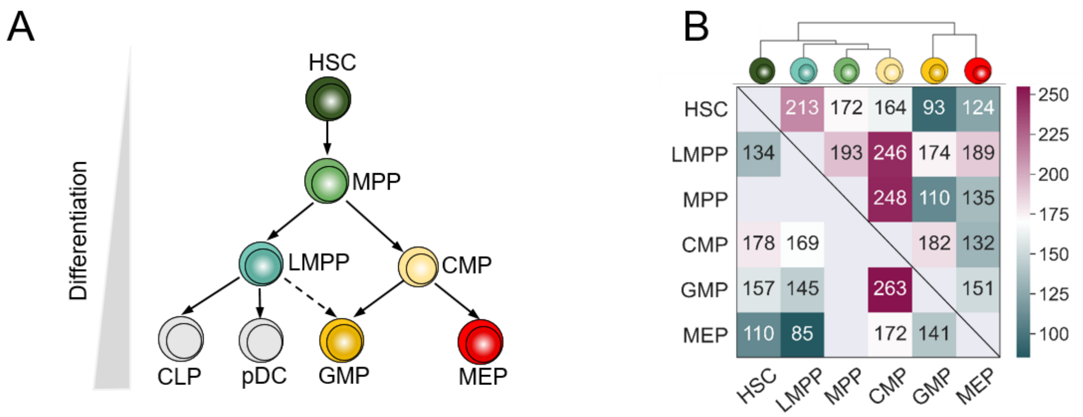

Fig 5. (A) A schematic of mouse hematopoietic differentiation. The cells in gray color cell type were not present in our datasets. (B) Heat map shows the number of similarly expressed genes selected by ADC cross six cell types (FDR=0.10). The upper triangular is the result of mouse hematopoietic cells while the lower triangular is the result of human hematopoietic cell data downloaded from GEO with accession code GSE117498. Unsupervised hierarchical clustering analysis was performed with the reciprocal value of the number.

6. Another interesting question the authors haven't explore is cross-species conservation. Can the proposed method reveal genes that have similar expression patterns between human and mouse?

Response: This is a good suggestion. We applied ADC to the hematopoietic stem cells (HSCs) data between human (Pellin, 2019) and mouse (Nestorowa, 2016) to find tissue-specific conserved genes. The annotation information of homologous genes was obtained by R package biomaRt (Smedley, 2009). Cells with less than 200 expressed genes and genes expressed in less than 3 cells were filtered. Also, cells with more than 25% mitochondrial genes present were filtered. After that, we normalized the gene counts per

cell with the scanpy package and log-transformed the expression with a pseudo-count 1 and the z-score normalization was also performed for each gene. ADC returned 232 similarly expressed genes where FDR was set as 0.01. The enrichment analysis was done by metascape (Zhou, 2019), which shows that these genes capture conserved functions between human and mouse, and these functions also exist in HSCs. Specifically, signaling by GPCR (G protein-coupled receptor) is the top one enriched function, where GPCRs are widely expressed in human and mouse (Demetrios, 2003). Also, GPCRs are wildly expressed in HSCs (Mohle, 2012). We added one related sentence to the Discussion section (page 14, line 328-330) and the related results to the Supplementary Materials (page 4, line 11-23):

"Also, ADC find tissue specific genes which are conserved across human and mouse, where these genes captures conserved functions across these species (Supplementary Fig S6)."

"We applied ADC to the hematopoietic stem cells (HSCs) data between human (Pellin, 2019) and mouse (Nestorowa, 2016) to find tissue-specific conserved genes. The annotation information of homologous genes was obtained by R package biomaRt (Smedley, 2009). Cells with less than 200 expressed genes and genes expressed in less than 3 cells were filtered. Also, cells with more than 25% mitochondrial genes present were filtered. After that, we normalized the gene counts per cell with the scanpy package and log-transformed the expression with a pseudo-count 1 and the z-score normalization was also performed for each gene. ADC returned 232 similarly expressed genes where FDR was set as 0.01. The enrichment analysis was done by metascape (Zhou, 2019), which shows that these genes capture conserved functions between human and mouse, and these functions also exist in HSCs. Specifically, signaling by GPCR (G protein-coupled receptor) is the top one enriched function, where GPCRs are widely expressed in human and mouse (Demetrios, 2003). Also, GPCRs are wildly expressed in HSCs (Mohle and Drost, 2012)."

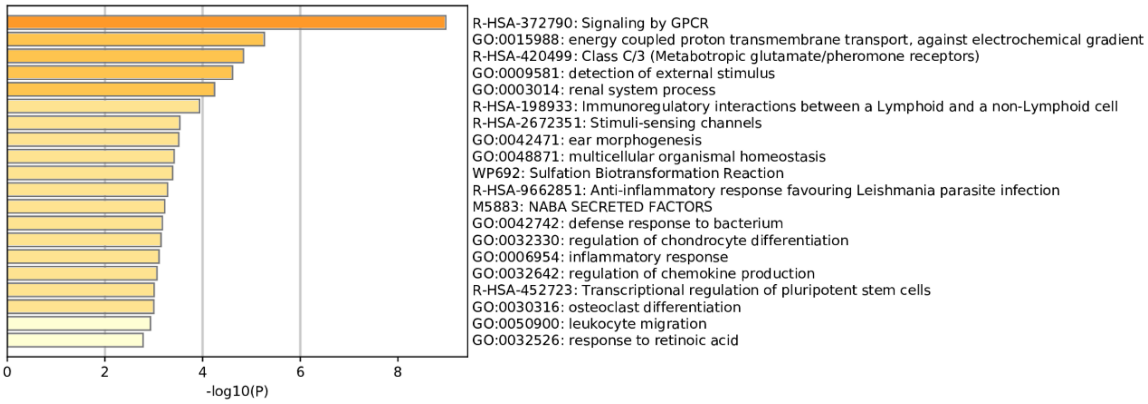

Supplementary Fig S6. Enriched GO terms of similarly expressed genes between human and mouse done by metascape.

[1] Pellin D, Loperfido M, Baricordi C, et al. A comprehensive single cell transcriptional landscape of human hematopoietic progenitors[J]. Nature communications, 2019, 10(1):

1-15.
[2] Nestorowa S, Hamey F K, Pijuan Sala B, et al. A single-cell resolution map of mouse hematopoietic stem and progenitor cell differentiation[J]. Blood, The Journal of the American Society of Hematology, 2016, 128(8): e20-e31.
[3] Zhou Y, Zhou B, Pache L, et al. Metascape provides a biologist-oriented resource for the analysis of systems-level datasets[J]. Nature communications, 2019, 10(1): 1-10.
[4] Vassilatis D K, Hohmann J G, Zeng H, et al. The G protein-coupled receptor repertoires of human and mouse[J]. Proceedings of the National Academy of Sciences, 2003, 100(8): 4903-4908.
[5] Möhle R, Drost A C. G protein–coupled receptor crosstalk and signaling in hematopoietic stem and progenitor cells[J]. Annals of the New York Academy of Sciences, 2012, 1266(1): 63-67.
[6] Smedley D, Haider S, Ballester B, et al. BioMart–biological queries made easy[J]. BMC genomics, 2009, 10(1): 1-12.

7. For Figure 7, the authors studied the similar genes between PBMC scRNA-seq and scATAC-seq datasets. Recently, multi-modal scRNA-seq+scATAC-seq datasets are also available for PBMC which can be obtained from the 10X genomic website. It will be interesting to see if the genes identified by the proposed method between scRNA-seq and scATAC-seq are similar to those identified using simple correlation metrics based on the multi-modal datasets.

Response: This is a good suggestion. We applied ADC to such multi-modal datasets. We downloaded such a multi-modal dataset from https://support.10xgenomics.com/single-cell-multiome-atac-gex/datasets/2.0.0/pbmc_granulocyte_sorted_3k which contains scRNA-seq and scATAC-seq data for each cell. To compared the similarities of genes between these two modalities, we first generated a gene activity matrix where each gene is quantified by scATAC-seq counts in the 2 kb-upstream region and gene body, using the GeneActivity() function in the Signac package. We filtered cells with less than 200 genes expressed and genes expressed in less than 3 cells. After that, we got 2685 common cells and 14056 common genes between these two data. We next calculated log(cpm(counts)+1) and perform z-score normalization for the gene expression matrix and gene activity matrix and would like to compared Pearson correlation coefficient (PCC) with ADC. It worth noting that the sparsity of the gene expression matrix of scRNA-seq and the gene activity matrix of scATAC-seq is extremely unbalanced (94.74% vs 67.16%), which may prevent us from uncovering true intergenic correlations.

We selected 195 similarly expressed genes by PCC > 0.3 and use 0.02 as FDR threshold to select 208 similarly expressed genes. The enrichment analysis was done by metascape (Zhou, 2019). We found only two genes (i.e., AGO4 and CD86) intersected between two sets of similar expressed genes. However, it's interesting that the two set of genes both reflects functions of PBMCs, e.g., the top first function of the genes selected by PCC is regulation of gamma-delta T cell differentiation (Fig A). The functions of genes selected by ADC also show PBMC related biological process and pathways (Fig B), such as regulation of T-helper 2 cell differentiation, Axon guidance (Boneschansker, 2016), positive regulation

of protein phosphorylation (Huang, 2021). It is interesting that these two set of similarly expressed genes act in complementary ways since we can see many intersections between these gens (Fig C).

We further looked at the intersection of the top 500 genes selected by PCC and ADC, where we found 10 common genes: AC108925.1, AGO4, C1orf162, CD86, ERCC8, MGST1, NFE2L3, OPRM1, RSF1, TNPO1, most of which are strongly PBMC-related (Uhlen, 2020). Also, if we looked at the intersection of the top 1000 genes selected by PCC and ADC, we will find 49 common genes (AC007163.1, AC108925.1, ACAD9, ACCS, ACSL3, AGO, ARHGAP21, C1orf162, CD86, CLHC1, CWC15, EFCAB12, EIF5A2, ERCC8, GALNT6, GRK7, IRF2BP2, JMY, KBTBD6, KCP, LTB, MARC1, MGST1, MMEL1, MOG, MRPL43, NADSYN1, NFE2L3, NUDCD2, OPRM1, PADI6, PDZK1, PHF1, 'PIGV, PPID, RAPGEF2, RNASET2, RPL29, RRH, RSF1, SEMA4D, SLC16A9, SLC4A8, SMIM15, SQLE, TNPO1, TRIM3, TYW5, WDR78) and most of them are related with PBMC.

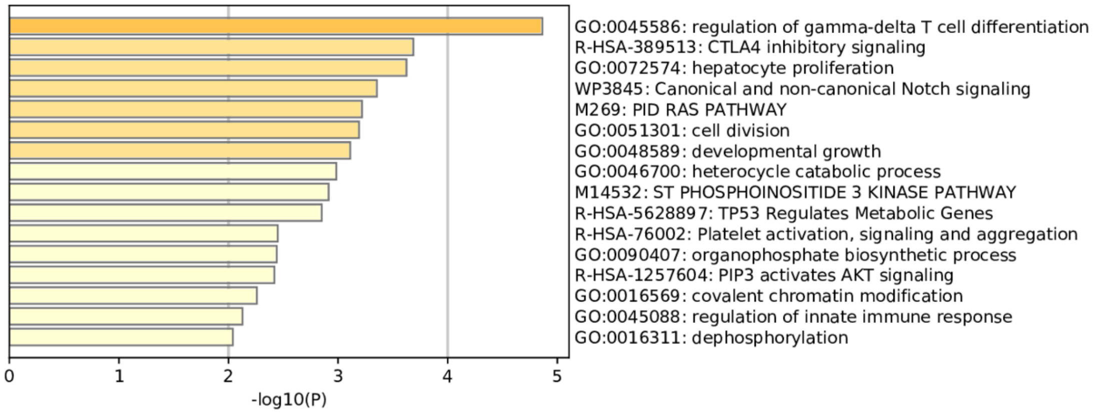

Fig A. The enriched functions and pathways of 195 genes selected by PCC>0.3.

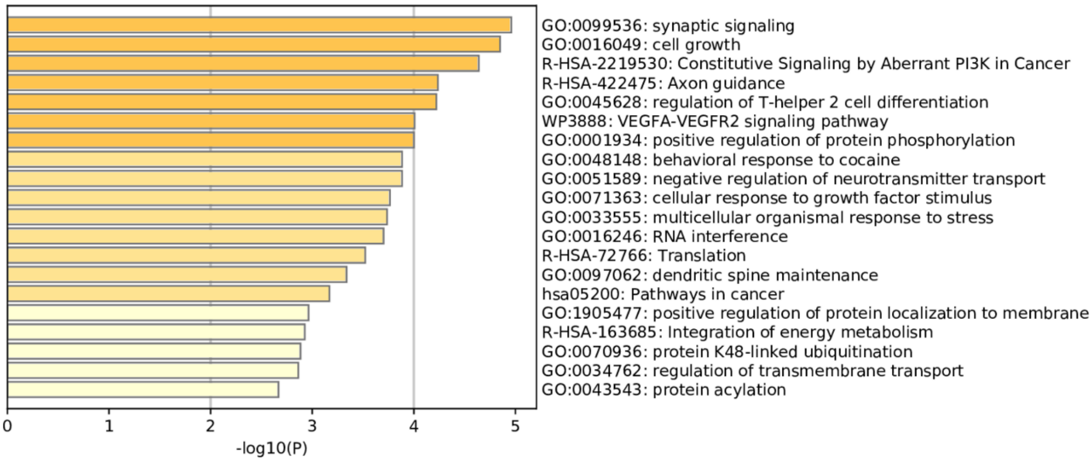

Fig B. The top 20 enriched functions and pathways of 208 genes selected by ADC with FDR<0.02.

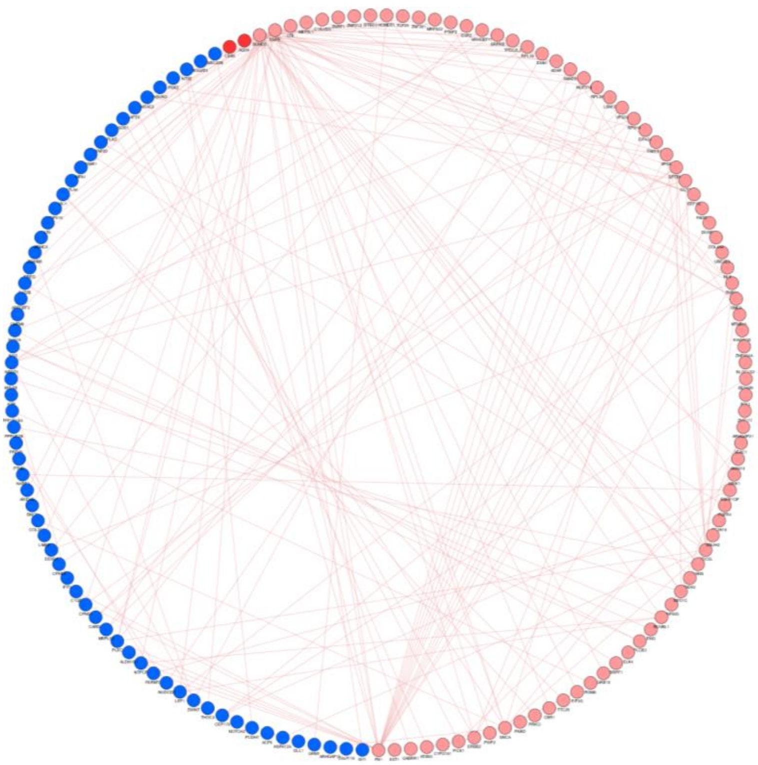

Fig C. The PPI network of the genes selected by ADC with FDR<0.02 (pink), PCC > 0.3 (blue) and both of these methods (red), which is plotted by Cytoscape (Smoot, 2011), genes with no intersections were removed.

[1] Zhou Y, Zhou B, Pache L, et al. Metascape provides a biologist-oriented resource for the analysis of systems-level datasets[J]. Nature communications, 2019, 10(1): 1-10.

[2] Boneschansker L, Nakayama H, Eisenga M, et al. Netrin-1 augments chemokinesis in CD4+ T cells in vitro and elicits a proinflammatory response in vivo[J]. The Journal of Immunology, 2016, 197(4): 1389-1398.

[3] Huang S, Zheng F, Liu L, et al. Integrated proteome and phosphoproteome analyses of peripheral blood mononuclear cells in primary Sjögren syndrome patients[J]. Aging (Albany NY), 2021, 13(1): 1071.

[4] Uhlen M, Oksvold P, Fagerberg L, et al. Towards a knowledge-based human protein atlas[J]. Nature biotechnology, 2010, 28(12): 1248-1250.

[5] Smoot M E, Ono K, Ruscheinski J, et al. Cytoscape 2.8: new features for data integration and network visualization[J]. Bioinformatics, 2011, 27(3): 431-432.

8. The application of the proposed method to data integration is not convincing. The state-of-the-art methods for single-cell data integration such as Harmony are not affected by the order of sample in integration. Also, the example is too simple. If the authors want to show the usage of their method in integration, they may want to provide a more comprehensive analysis.

Response: The main contribution of ADC is to select similarly distributed genes, the number of which can show the relative similarity of two datasets. That is true that some of

the state-of-the-art methods are not affected by the order of sample in integration such as Harmony. But some methods like MNN (Haghverdi, 2018), Seurat (Butler, 2018) indeed rely on the order of the datasets. In light of your comment, we removed this toy example, which doesn’t affect our main conclusion.

[1] Haghverdi L, Lun A T L, Morgan M D, et al. Batch effects in single-cell RNA-sequencing data are corrected by matching mutual nearest neighbors[J]. Nature biotechnology, 2018, 36(5): 421-427.
[2] Butler A, Hoffman P, Smibert P, et al. Integrating single-cell transcriptomic data across different conditions, technologies, and species[J]. Nature biotechnology, 2018, 36(5): 411-420.

Response to Reviewer #2

In this paper, the authors propose to use the Approximate Distance Correlation (ADC) to identify similarly distributed genes between two biological datasets, and subsequently to use the identified genes to measure the similarity between these two datasets. For each gene, they first identify its k most correlated genes within each dataset, and then they use the measurements of these two sets of k genes in their corresponding datasets to calculate the distance correlation (DC), which they define as the ADC. Although the paper is overall clearly written, we have several major concerns about the validity of the methodology. In our opinion, these concerns, if not addressed, will preclude ADC to be a useful data analysis tool.

1. Our first concern is the motivation of ADC. If the goal is to identify genes with similar distributions in two datasets, then what is the definition of “distributions.” To us, this is the key and should be clarified in the beginning.

Response: The definition of “distribution” here is that the expression of a gene across a given number n of cells was considered as the n expression observations of this gene in this dataset. Since the cells among two datasets here cannot be matched. So ADC measures the expression distributions of a gene, which doesn’t rely on the order of the cells. To clarify this, we added one sentence to the Introduction (page 3, line 3-5):

“The distribution here indicates that the expression of a gene across a given number n of cells was considered as the n expression observations of this gene in this dataset.”

2. We have a major concern about applying the distance correlation to the manually selected k genes in each dataset. In Section “Materials and methods”---"Select approximate observation for each gene," distance correlation requires (X_i, Y_i) to be a true pair, and {(X_i, Y_i)} to be iid. Here, however, (X_i, Y_i) is not from the same gene and thus not naturally paired, and {(X_i, Y_i)} are neither independent (they are manually selected, with high correlation) nor identically distributed (they are from different genes). Hence, the basic assumptions of the distance correlation are violated.

Response: Our main motivation is to find similarly distributed genes between two datasets. This is an important issue as we have demonstrated in four real applications. However, multiple observations for each gene are required to achieve it. But this is unrealistic. If we indeed have multiple IID observations, then these observations must be highly correlated. That is why we manually selected a give number of highly correlated genes as an approximation to solve this challenging task.

3. Due to the fact that there exists only one observation for each gene, we suggest that the authors view the biological problem on a metagene level as a remedy. To be more specific, authors can first cluster genes into metagenes in the original datasets, and then they can calculate the distance correlation for each metagene. In this way, replicates (X_i, Y_i) are naturally paired for the i-th gene in metagene.

Response: In this way, for many genes in a cluster pair, they will have the same distance correlation. So their similarity will be indistinguishable. This makes it fail to capture the degree of similarity at single-gene resolution.

4. If the authors do want to achieve the single-gene resolution, to remedy the violation of the assumptions required by the distance correlation, the authors may consider taking the advantage of gene orthology information instead of using the same data twice to select the top k correlated genes for every gene. At least the authors must make sure that (X_i, Y_i) is paired by external information, not the data to be used to calculate the distance correlation.

Response: Since we didn't focus on cross species, we are confused about the "gene orthology". We supposed you mean gene ontology information. In this way, for all the genes in a GO term, they will have the same distance correlation. So their similarity will be indistinguishable. Thus, it is infeasible.

5. There's little discussion on the choice of parameter k (number of selected highly correlated genes in each dataset). It affects the sets of genes for distance correlation calculation, as well as the asymptotic distribution. Instead of simply proposing a default value of k, the authors should discuss the effect of k and provide guidance on choosing k.

Response: We have performed a simulation study to justify the selection of k (page 8, line 218-222): "We also test the influence of hyper-parameter k by applying ADC to Data 1 and Data 2. Here, we selected the top 100 similarly expressed gene under each k varying from 20 to 40. For each pair of k, the number of overlapped genes is calculated. In average, we got almost 93 genes, that suggests ADC is a robust algorithm considering the selection of k (Supplementary Fig. S2C)."

In light of your comments, we also proposed a more robust process to select the similarly expressed genes. Specifically, we detected the similarly expressed gene under each k varying from 20 to 40, and we kept genes which were considered as similarly expressed ones under 21 different ks. We got very consistent result on real biological applications as the original process (e.g., for the "Similarly correlated genes across modalities reveal biological relevant genes" applications in Fig. 6B and Supplementary Fig. S5E).

To clarify this, we added some sentences to the conclusion (Page 14, line 332-339).

"The number k of selected highly correlated genes in each dataset is an important parameter which could affect the gene sets for DC calculation. We have empirically demonstrated the default one work well in the simulation studies and biological applications. Alternatively, we could provide a more robust version by leveraging the similarly expressed genes selected by ADC with different ks. Specifically, we detected the similarly expressed

gene under each k varying from 20 to 40, and we kept genes which were considered as similarly expressed ones under 21 different ks. The results are very consistent with the original ones (Fig. 6B and Supplementary Fig. S5E)."

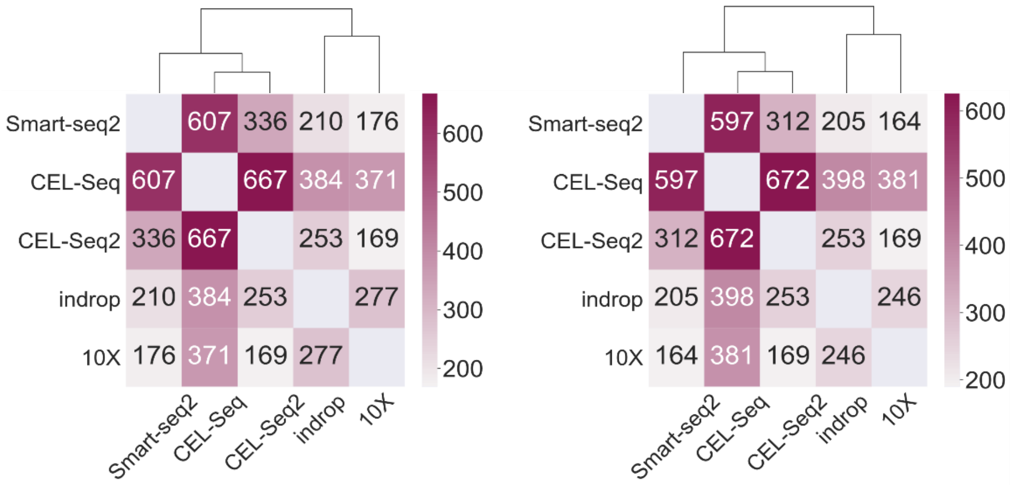

Fig. 6B and Supplementary Fig. S5E. Similarly expressed gene selected by ADC with k=30 (left) and an ensemble version (right) between each pair of these datasets (FDR=0.01). Unsupervised hierarchical clustering was performed with the reciprocal value of the number in both situations.

6. In Fig 6A, we see that results are far from satisfactory in the overlap of top HVGs. We wonder whether this is a fair comparison. Are the selected top HVGs restricted to the common genes shared between datasets?

Response: In light of your suggestions, we selected top HVGs restricted to the common genes shared between datasets. Our original conclusion still stands, i.e., the number of intersected HVGs failed to reveal the relative differences of these technologies (Fig 6).

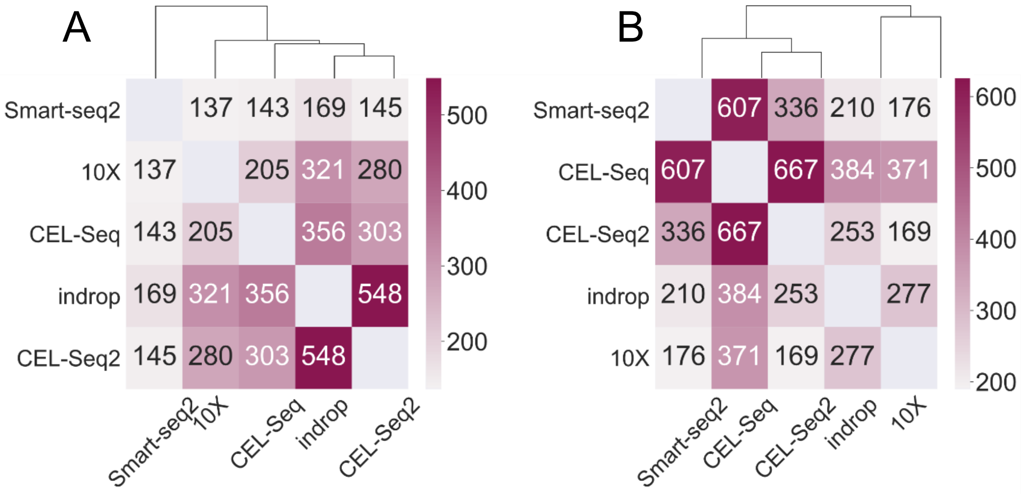

Figure 6. (A) Number of overlapping genes among the top 1000 HVGs of the data from five different technologies, and (B) similarly expressed gene selected by ADC between each

pair of these datasets (FDR=0.01). Unsupervised hierarchical clustering was performed with the reciprocal value of the number in both situations.

7. The authors should apply more direct validation other than comparing the number of similarly expressed genes and the data structure. For example, to highlight that the algorithm can detect similarly distributed genes across datasets, the authors can directly compare the overall expression pattern of the same genes with high ADC scores in both datasets.

Response: Yes, to demonstrate the effectiveness of ADC, we applied it to four diverse biological applications with enrichment analysis and gene network. Logically, if we can directly measure the distribution similarity of any two genes, that will be an easy task. Actually, this is unrealistic. There is only very weak similarity among two any genes. That’s why we develop ADC to achieve it.

Response to Reviewer #3

In this manuscript, the authors develop a statistical method, namely Approximate Distance Correlation (ADC) for detecting genes with similar expression patterns across multiple samples or datasets. ADC is an alternative to DC, where multiple samples are needed for calculation. Instead of multiple samples, ADC utilizes the genes that are highly correlated with a gene, as alternative observations. The authors formulate their solutions and show its effectiveness with multiple case studies.

Although the idea seems interesting and the case study results are impressive, there are some problems with the manuscript, as pointed out below:

1. The most important issue about this method is using the correlated genes as alternative observations for a gene. Authors tell that this is necessary because often the experiment cannot be repeated for the same sample, noting that "Profiling the given cells for multiple times to achieve this is unrealistic, since cells are destroyed during the measurement process." However, in the same manner, using correlated genes as alternative observations is similarly unrealistic, since almost every gene has its own distinct expression pattern. In this sense, I think it is highly questionable to use correlated genes for this purpose, instead of relying on multiple cells, which are usually in the same biological state. I think the approach followed here, using ADC instead of DC, is only diverting the problem of reproducibility on a different aspect, instead of solving it.

Response: We feel sorry we didn't clearly state our contribution. Our main motivation is to find similarly distributed genes between two datasets. This is an important issue as we have demonstrated in four real applications. Since we don't have multiple observations for each gene, DC cannot be used for our task directly. The reason is that for a given gene, we cannot have multiple observations for one cell (not multiple cells). That is why we manually selected a give number of highly correlated genes as an approximation to solve this challenging task.

Note there is a limited number of cell populations for a given tissue. Mathematically, this indicates that the expression matrix (gene vs cell) is a low-rank one. Thus, the expression of some genes should be highly correlated and there is a limited number of gene patterns. It is not unrealistic that almost every gene has its own distinct expression pattern.

2. In this context, the authors should compare their method to using Distance Correlation (DC) for each benchmarking and case study. Does their method (ADC) outperform DC, by using the same set of samples? Since ADC depends on correlation, which requires multiple samples or cells, DC can be computed and used for every test.

Response: The DC cannot measure the gene similarity across datasets directly. For DC, if we view genes as one-dimensional variables, then we need the cells across datasets are matched (just like Pearson correlation coefficient). If we view genes as high dimensional variables, we need multiple observations for each gene. However, profiling the given cells for multiple times to achieve this is unrealistic, since the cells are destroyed during the

measurement process, that's why we develop ADC (see Introduction).

3. Page 5, Line 117: "Profiling the given cells for multiple times to achieve this is unrealistic. Since cells are destroyed during the measurement process" should be single sentence "Profiling the given cells for multiple times to achieve this is unrealistic, since the cells are destroyed during the measurement process."

Response: In light of your suggestions, we have revised this sentence (Page 5, line 119-120):

"*Profiling the given cells for multiple times to achieve this is unrealistic, since the cells will be destroyed during the measurement process.*"

4. Page 7, Line 176: What do m and n denote? Genes and cells?

Response: Here, m and n denote the dimension of the simulated variables X and Y, they have no practical meaning since they are just random variables drew from random distributions. We have made detailed description for m and n (Page 7, Line 178):

"The dimensions of each pair of X and Y are m=3000 and n=10000, respectively."

5. Page 8, Line 216: "Although there are only 40% cells overlapped between Data 1 and Data 3" contradicts with the Fig. 3 legend: "40% of cells in Data 1 and Data 2 are of the same type." Is it Data 2 or Data 3?

Response: Yes**,** you are right. We have corrected this typo.

6. Page 9, Figure 3 legend: FDR is chosen 0.20? Why so large cutoff? Generally FDR is chosen 0.01 or 0.05, very rarely 0.1 at most.

Response: It is reasonable to choose FDR as 0.20, while many FDR controlling methods knockoff (Candes, 2020), PC screen (Liu, 2021) use 0.20 or even bigger as a cutoff, to increase the power, use a little bigger FDR threshold is acceptable. In light of your comment, we choose FDR as 0.05, and use the results under this threshold to replace the origin one. However, our results still hold, i.e., the number of similarly expressed genes reflect the similarities between these datasets.

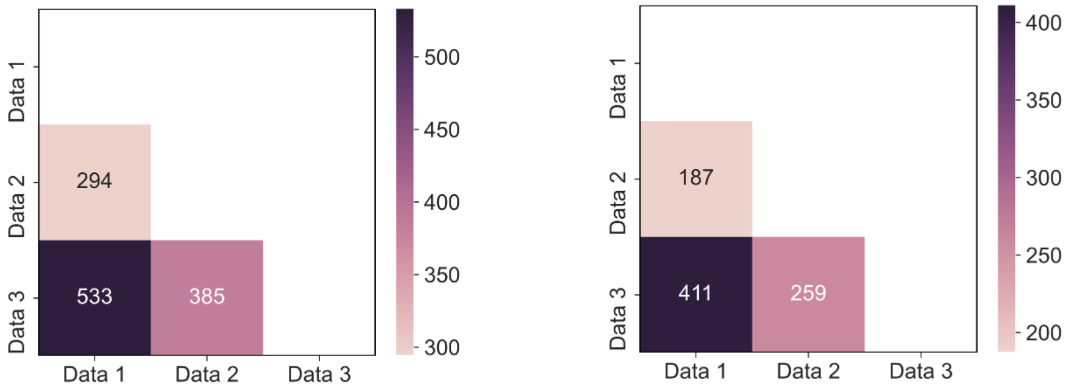

Origin Fig3.B and New Fig.3B. Heat map of the similarly expressed genes selected by ADC across Data 1, Data 2 and Data 3 (FDR=0.20) and (FDR=0.05).

[1] Candès E, Fan Y, Janson L, et al. Panning for gold:'model - X'knockoffs for high dimensional controlled variable selection[J]. Journal of the Royal Statistical Society: Series B (Statistical Methodology), 2018, 80(3): 551-577.
[2] Liu W, Ke Y, Liu J, et al. Model-free feature screening and FDR control with Knockoff features[J]. Journal of the American Statistical Association, 2020: 1-16.

7. Page 9: The authors do some GO annotation for similar genes and find cancer hallmark terms as a result. Using DC or just overlapping highly expressed genes can also give the same results. Is ADC superior in any sense? And just finding hallmark terms doesn't mean ADC could gain deep insights, since this is nothing new. It is just some kind of validation.

Response: As we replied to your **Question 2**, DC cannot measure the gene similarity across datasets directly. The DC cannot measure the gene similarity across datasets directly. For DC, if we view genes as one-dimensional variables, then we need the cells across datasets are matched (just like Pearson correlation coefficient). If we view genes as high dimensional variables, we need multiple observations for each gene. However, profiling the given cells for multiple times to achieve this is unrealistic, since the cells are destroyed during the measurement process, that's why we develop ADC (see Introduction). The selection of highly expressed genes only considers the information of second moment, while ADC considers arbitrary moments by characteristic functions, which includes more information. As we have showed in different application scenarios, ADC is more flexible. For example, the number similarly expressed genes can reflect the similarities across technologies while highly variable genes cannot (Fig 6).

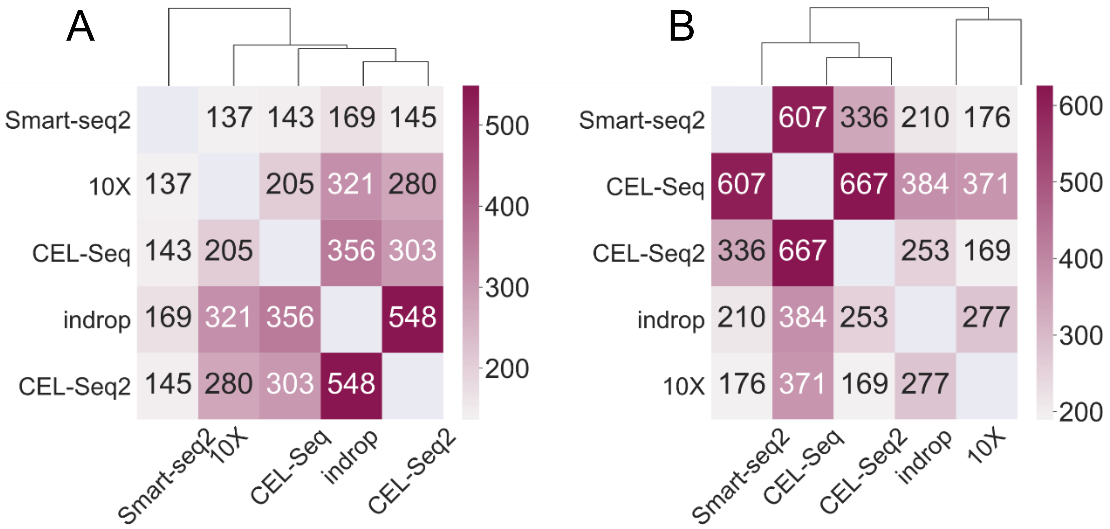

Fig 6. (A) Number of overlapping genes among the top 1000 HVGs of the data from five different technologies, and (B) similarly expressed gene selected by ADC between each pair of these datasets (FDR=0.01). Unsupervised hierarchical clustering was performed with the reciprocal value of the number in both situations.

8. Page 10: Figure 4 C and E. In the heatmap, hundreds of genes are reported but very small number of genes are shown in C and E. How are those genes selected? Why is particularly special about those genes? What are the node sizes proportional to? Why are some genes colored pink and others white?

Response: Originally, we have sated the details of the network analysis in Supplementary file (page 3) "**1.7. Functional network analysis of the selected genes**". We feel sorry that we didn't clarify it clearly. In light of your comments, we added all the details to Supplementary Materials (Page 3, line 41):

"We analyzed the functional network of the top selected genes by GeneMANIA. The input genes were showed in red colour and top 10 related genes were added into the network which were showed in white colour. Physical interactions and genetic interactions were showed with different coloured edges. The size of the node is proportional to its degree, and the network was plotted by Cytoscape. Unless carefully specified, we use top 100 similarly expressed genes selected by ADC as the input, the subnetwork which only contains less than 3 genes were removed in the output of GeneMANIA."

9. Page 11. Figure 5A. What are those cell types (HSC, MPP, etc.)? Since not every reader is a hematologist, these acronyms should be defined in the figure caption.

Response: Originally, we have explained these cell types (Page 3, line 68-71): "There are six cell types remained including hematopoietic stem cells (HSC), common myeloid progenitors (CMP), granulocyte-onocyte progenitors (GMP), megakaryocyte-erythroid progenitors (MEP), common lymphoid progenitor (MPP), lymphoid-primed multipotent

progenitor (LMPP) with 323, 328, 123, 362, 368, 280 cells, respectively."

10. Page 12, Line 287 and Fig. 6: "Thus, top HVGs of the five datasets are supposed to have many overlaps since they were all profiled about the similar cell types. However, we only found a few HVGs overlapped among these datasets (Fig. 6A)." The numbers in the heatmaps in Fig 6A and 6B are not comparable. If you start with 1000 genes for an analysis and 20,000 genes for the other, you will obviously get different numbers in total. The actual numbers are not comparable between two. Only clustering can be compared.

Response: In light of your comment, we removed this sentence.

11. Page 13, Figure 7C: Similar issue. Like Fig 4 C and E, how are those genes selected? Why is particularly special about those genes? What are the node sizes proportional to? Why are some genes colored pink and others white?

Response: Originally, we have sated the details of the network analysis in Supplementary file (page 3) "**1.7. Functional network analysis of the selected genes**". We feel sorry that we didn't clarify it clearly. In light of your comments, we added all the details to the Supplementary Materials (page 3, line 41) (See response to **Question 8**).

12. Although the authors developed a method, they did not provide the computational implementation of their framework in any platform. In this context, this study has little benefit for the scientific community. The authors must compile a software package in a widely used open-source programming language (such as R) not a commercial software language (such as Matlab), so that any researcher interested in this method and can freely download and apply this method on their own data. The software must be deposited in a publicly accessible repository such as GitHub and properly documented with code comments, package reference and tutorials. Any researcher should be able to download and apply it in a couple hours without need to intensive effort.

Response: We have uploaded our code to (https://github.com/zhanglabtools/ADC), our code is implemented by Python, which is an open-source programming language. We have provided detailed code comments, package reference and tutorials. As we have discussed that, even for two datasets of 1,000,000 cells and 10,000 genes, ADC can be finished in 2.2 hours. We added the following sentence (page 6, line 160)

"ADC was implemented in Python and the package can be downloaded from https://github.com/zhanglabtools/ADC."

13. In addition to publishing the software package for the framework, the authors should compile a separate package for just re-producing all the results presented in this manuscript. The authors must provide all the code, scripts and documentation in

addition to interim data to replicate the results presented. If the public datasets that are used is reasonable size, the authors should also deposit it in a repository for easy access. If some of the public data is very large (larger than > 100GB), the authors should provide links to download the data with information about how to process it.

Response: In light of your suggestion, we compile a separate package for just re-producing all the results in https://github.com/zhanglabtools/ADC. As written in the main manuscript, we provided specific links to download the data and detailed information in it about how to process them (page 6, line 160):

"ADC was implemented in Python and the package can be downloaded from https://github.com/zhanglabtools/ADC."
