# Supplementary material for: Approximate distance correlation for selecting highly interrelated genes across datasets
Source: PLoS Comput Biol. 2021 Nov 9;17(11):e1009548. doi: 10.1371/journal.pcbi.1009548 (PMC8604336; doi:10.1371/journal.pcbi.1009548)
Supplement: Attachment — Submitted filename: ReviewReport.pdf [file pcbi.1009548.s003.pdf]

Response to Reviewer 2

Reviewer #2: The authors did not address the major concerns raised by Reviewers 2 and 3 regarding the validity of the proposed method ADC. Most importantly, the biological meanings of the genes selected by ADC are hard to interpret. Moreover, we do not agree with the authors that these genes should be referred to as "similarly distributed genes." We did a simulation study to show that a gene with the same distribution in two batches of cells cannot be captured by ADC. Hence, we are not convinced that ADC has a solid probability foundation or a biological relevance.

Response: We appreciate very much for your kind comments, and we feel sorry that we didn't well clarify our method clearly. The concept of "similarly distributed genes" may mislead you. In the following, we explain our revision on it and clarify why ADC is a valid method.

Below please find our detailed comments.

1. The biological meanings are unclear for the "similarly distributed genes" identified by ADC. The current ADC implementation tries to do the following. Take k = 4 as a toy example; for every tested gene, it selects (k-1) top correlated genes in both datasets, which may be {A, B, C} in dataset 1, and {D, E, F} in dataset 2; ADC will identify this tested gene as a "simiarly distributed gene" if and only if {tested gene, A, B, C} and {tested gene, D, E, F} are "correlated" in the sense of distance correlation (which roughly means that the 6 pairwise distances among {tested gene, A, B, C} and the 6 pairwise distances among {tested gene, D, E, F} are correlated). However, there is no guaranteed correspondence between {A, B, C} and {D, E, F}, and thus the distance correlation between {tested gene, A, B, C} and {tested gene, D, E, F} is biologically meaningless. Also, how does this correlation inform whether the tested gene is "similarly distributed" or not? Please see point 2 below.

Response: You are right about the implementation of ADC. We agreed that there is no guaranteed correspondence between {A, B, C} and {D, E, F} for a tested gene. This is exactly what we employed in ADC to distinguish some genes between two datasets are interrelated and others are not.

Let's reconsider the original problem. Given two single-cell omics datasets (gene vs cell) of the same or related biological tissue (or system), some genes across them must be biologically related. We know that there is a limited number of cell populations for a given tissue. Thus, there are limited number of cell clusters for these two datasets. Mathematically, this indicates that the expression matrix (gene vs cell) is approximately a low-rank one. Thus, the expression of some genes should be highly correlated and there is a limited number of gene patterns in each dataset, which is related with a certain cell population.

For a tested gene, if it has a set of highly correlated genes (in a gene pattern) in both datasets respectively, that the DC between them is strong indicates the two gene sets have **biologically functional correspondence**. On the other hand, for a tested gene, if it only has a set of weakly correlated genes in one or both datasets respectively, **that the DC between them is relatively weak indicates they are not functionally related**.

ADC is a kind of correlation metric. It reflects the association strength of two genes here. The most significant ones capture the genes that has the most similar biological signals of the two data sets. Here the concept "similarly distributed" may mislead you (please see response to point 2 below). According to the above analysis, we changed it into “interrelated”.

2. There is a gap between what ADC claims to identify and what ADC actually identifies. What ADC claims to identify are the genes that have similar distributions in two datasets, where they have m and n observations, respectively. ADC aims to perform the following hypothesis testing: for every tested gene, X \in R^m, Y \in R^n are 2 random vectors, representing that gene's count vectors in m and n cells, respectively; H_0: X and Y are independent vs. H_a: X and Y are not independent" (Page 5, Section "DC"). While ADC claims/aims to identify those genes that reject H_0, what ADC actually does is different (please refer to our comment 1 that ADC actually identifies those genes whose top correlated genes are "correlated" between the two datasets). In the claim, X and Y should both correspond to the tested gene; however, in the actual implmentation, X refers a not-well-defined random vector whose four observations are {tested gene, A, B, C}, while Y refers a not-well-defined random vector whose four observations are {tested gene, D, E, F}. Given that A, B, and C are selected based on their high correlations with the tested gene in the first data set, it is impossible to assume that {tested gene, A, B, C} are i.i.d. observations of X. Hence, the actual implmentation of ADC is invalid in the probability sense.

We design the following simulation to demonstrate this gap:

Generate X1, X2, ..., Xm, Y1, Y2, ..., Yn ~ N(0, I_p) i.i.d.
Denote X = [X1, X2, ..., Xm], Y = [Y1, Y2, ..., Yn], where rows correspond to the p genes, and the m and n columns correspond to the m and n cells in two datasets.
Run the python code provided by the authors.
In the result, ADC does not identify any similar genes (FDR = 0.05).
This result is expected because for every tested gene, the top k correlated genes in X and Y are independently simulated and thus not correlated, so no gene should be identified by the ADC method as implemented. ("the actual goal")
However, by design, every gene is simulated from the same standard Gaussian

distribution in the two datasets, so it perfectly satisfies the definition of "similarly distributed gene" and thus should be identified. ("the ideal goal")

Therefore, we conclude that the actual goal of ADC is different from its ideal goal.

Response: You are right about the actual goal of ADC. ADC is indeed a heuristic/approximate DC algorithm which doesn't strictly assume that {tested gene, A, B, C} are i.i.d. observations of X like DC. This is why it is named as approximate DC (ADC).

In your simulation study, two simulated datasets which independently sampled from a normal distribution. Obviously, there should be no genes selected by ADC between them. The application is correct and it demonstrated that ADC could work well. Besides, even for n=m, and the samples of X and Y are matched, we still cannot expect to discovery correlation even their distribution is the same one across X and Y by the Pearson correlation coefficient.

We didn't well clarify the concept "similarly distributed gene", which may mislead you. "similarly distributed" doesn't mean "same distribution". Our ideal goal is to find genes than shared some correlated (dependent) structure across datasets. Obviously, the two genes simulated from the same standard Gaussian distribution don't mean they are correlated. In other words, even they are sampled from the same distribution, they are independent (not correlated).

In our simulation studies (e.g., **Scenario 1)**: Draw $X = (x_1, \cdots, x_m)^T$ independently from a standard normal distribution. Let $y_i = sx_i$ (i=1,⋯,l) and draw y_i (i=l+1,⋯,n) independently from a standard normal distribution, where $s \sim U(1, 5)$. You could see what dependence means here. ADC can well detect such dependent ones. According to the above analysis, we changed "similarly distributed" into "interrelated" (see response to point 3 below).

3. To re-formulate ADC as a valid method, we deem it necessary for the authors to accurately and formally define "distribution" and "similar genes" using probability notations.

Response: Like distance correlation (DC), we aimed to quantify any types of dependence including non-monotone, non-linear dependence between two gene vectors across datasets. Similar with DC, the result of ADC is independent of the order of the gene vector dimensions (i.e., the order of the cells), which implies ADC captures the dependence of distributions. However, this doesn't mean that two gene vectors come from the same distribution. To prevent such misleading, we changed the term 'similarly distributed genes' to "interrelated genes".

ADC aims to perform the following hypothesis testing: for every tested gene, $X \in R^m$, $Y \in R^n$ are two random vectors, representing that gene's count vectors in m and n cells, respectively; H_0: X and Y are independent vs. H_a: X and Y are not independent" (Page 5, Section "DC"). Naturally, multiple observations for each gene across both datasets are needed to estimate the empirical characteristic functions. However, we usually only have one observation for each gene across pairwise biological omics data. Profiling the given cells for multiple times to achieve this is unrealistic, since the cells are destroyed during the measurement process. Thus, it is impossible to perform the estimation directly. As we know, a set of genes usually tend to be highly correlated under the same condition due to the modular organization of biological systems. In view of this, for a target gene, we introduce an approximate strategy to select k highly correlated ones with it as alternative observations in a single dataset to overcome this issue. We applied this procedure to all the genes individually (Page 5-6, Select approximate observations for each gene).

In light of your suggestion, we carefully define "distribution", changed the writing about "similarly distributed genes" and polished related writing. For example,

Page 2, line 28-35: *"Specifically, for a target gene, we would like to measure the correlation of this gene across datasets, i.e., the correlation between two gene vectors with different samples (dimensions). This is a tough task since we don't have matched samples. Thus, the correlation is not supposed to rely on the order of the samples (order-free), which means this correlation is determined by the distribution of genes. The distribution here indicates that the expression of a gene across a given number of cells are considered as the expression observations of this gene in a given dataset."*

4. A potential fix: for a tested gene, if its top k correlated genes in the two datasets are paired by external information (e.g., gene orthology), shared, or naturally paired, the ADC result may carry more biological meaning.

Response: ADC is a valid data-driven method which doesn't employ any external information in current settings. We agreed with the reviewer that employing external information may improve ADC. But the way that making the top k correlated genes $\{A_1, A_2, A_3,\cdots,A_k\}$ are paired has essential limitation. In this way, for each gene A_i in {A1, A2, A3,⋯} has the same top k correlated genes, then all the tested genes A_i will have the same DC. So, their similarity will be indistinguishable. Moreover, it is hard to image all genes have naturally paired top k correlated genes in two datasets. Thus, we need to think other way to employ external information to improve ADC.

In light of your suggestion, we added the following sentences to improve the paper (page 15, line 363-366):

“ADC is a valid data-driven method which doesn’t employ any external information now. It will be an interesting direction to consider external knowledge (e.g., gene ontology) to improve it in future.”
